# 235. Tracking Invasive Extraintestinal Pathogenic Escherichia coli Disease in Older Adults in a Community-Based Clinical Trial Setting

**DOI:** 10.1093/ofid/ofac492.313

**Published:** 2022-12-15

**Authors:** Joachim Doua, Miquel Ekkelenkamp, Stephen Ruhmel, Kimberly Leathers, Clinical Research Associate, Oscar Go, Ranee Chatterjee, Michal Sarnecki, Jan Poolman, Marc Bonten

**Affiliations:** Janssen Research & Development, Infectious Diseases & Vaccines, Janssen Pharmaceutica, Beerse, Antwerpen, Belgium; Department of Medical Microbiology, University Medical Center Utrecht, Utrecht, Utrecht, Netherlands; Janssen Research & Development, Titusville, New Jersey; Duke University, Durham, North Carolina; Janssen Research & Development, Titusville, New Jersey; Duke University, Durham, North Carolina; Janssen Research & Development, Janssen Vaccines, Bern, Bern, Switzerland; Bacterial Vaccines Discovery and Early Development, Janssen Vaccines & Prevention B.V., Leiden, Zuid-Holland, Netherlands; Julius Center for Health Sciences and Primary Care, University Medical Center, Utrecht, Utrecht, Netherlands

## Abstract

**Background:**

A vaccine developed to prevent invasive extraintestinal pathogenic *Escherichia coli* disease (IED) is being evaluated in a phase 3 clinical trial (NCT04899336). However, historical performance of similar clinical trials has been challenged by inefficient endpoint catchment. Here we report the EXPECT-1 (NCT04087681) study findings on the capture techniques for IED and hospital admissions in a community-based setting.

**Methods:**

EXPECT-1 was a prospective, observational study conducted in networks of 8 hospitals with surrounding primary care centers in North America, Europe, and Asia. Participants were followed for 1 year to track hospital admissions and occurrence of IED as reported by the primary care physician (PCP) or the participant. In 151 US participants, a geofence technology was used to track admissions to hospital areas. Participants were eligible if aged ≥ 60 years and if they did not have severe chronic conditions. Data were collected from October 24, 2019 until January 28, 2021. Study outcomes included incidence of IED and the rate of hospital admission.

**Results:**

In total, 4470 participants were enrolled: 68% were female, 74.4% were white, median age was 70 years, and 59.4% had a urinary tract infection (UTI) in the past 10 years. Four IED events were captured with a cumulative incidence (95% CI) of 0.09% (0.024–0.229%) and an incidence rate of 98.6 cases (25.6–248.4) per 100,000 person-years. The incidence increased to 0.15% (0.041–0.385%) and 164.4 (43.8–420) per 100,000 person-years among participants with a history of UTI. Of the 4 IED events, 2 were participant self-reports, 1 PCP report, and 1 report via follow-up phone call. Of all hospital admissions, 56.6% were captured by PCPs and 48.7% by participants’ self-reports. The Kappa statistic varied from 0.31–0.65, indicating fair to moderate agreement between PCP reports and participant self-reports. Geofencing captured 99% of > 800 entries and exits from hospital areas.

**Conclusion:**

The findings suggest that older adults with a history of UTI in the past 10 years may be a good target population for an efficacy clinical investigation on IED. PCP reports, participant self-reports, and geofencing can be promoted to track study endpoints in a community-based clinical trial setting.

**Disclosures:**

**Joachim Doua, MD, MPH**, Janssen: Employee|Janssen: Stocks/Bonds **Stephen Ruhmel, MPH**, Janssen: Employee **Oscar Go, PhD**, Janssen: Employee of Janssen Research & Development LLC. **Ranee Chatterjee, MD, MPH**, Bristol Myers Squibb: Grant/Research Support|Epigenomics: Grant/Research Support|Verily: Grant/Research Support **Michal Sarnecki, MD**, Janssen: Employee|Janssen: Stocks/Bonds **Jan Poolman, PhD**, Janssen: Janssen Vaccines & Prevention B.V.|Janssen: Stocks/Bonds **Marc Bonten, MD, PhD**, Astra-Zeneca: Advisor/Consultant|Janssen: Advisor/Consultant|Merck: Advisor/Consultant|Novartis: Advisor/Consultant.

